# Blood carnitine profiling on tandem mass spectrometry in liver cirrhotic patients

**DOI:** 10.1186/s12876-020-01190-6

**Published:** 2020-02-19

**Authors:** Hisamitsu Miyaaki, Hironori Kobayashi, Satoshi Miuma, Masanori Fukusima, Ryu Sasaki, Masafumi Haraguchi, Kazuhiko Nakao

**Affiliations:** 1 Department of Gastroenterology and Hepatology, Nagasaki University Graduate School of Biomedical Sciences, 1-7-1 Sakamoto, Nagasaki, 852-8501 Japan; 2 Department of Pediatrics, Shimane University Faculty of Medicine, 89-1 Enya, Izumo, Shimane 693-8501 Japan

**Keywords:** Carnitine fraction, Liver cirrhosis, Acylcarnitine, Tandem mass spectrometry

## Abstract

**Background:**

The level and profiles of blood free carnitine and acylcarnitines, obtained by acylcarnitine analysis using tandem mass spectrometry, reflect various metabolic conditions. We aimed to examine the level of free carnitine and acylcarnitines in liver cirrhosis patients by acylcarnitine analysis and determine the clinical and subjective factors associated with blood carnitine fraction levels in liver cirrhosis.

**Methods:**

We compared blood carnitine fractions in 54 liver cirrhotic patients to other laboratory test results and questionnaire answers.

**Results:**

In almost all patients, the blood levels of free carnitine (C0) and acetylcarnitine (C2) were within the normal reference range. However, in some patients, the levels of long-chain acylcarnitines, such as C16 and C18:1-acylcarnitine, were higher than the normal reference range. Liver function, assessed by Child-Pugh score, was significantly correlated with the blood level of each carnitine fraction measured (C0, C2, C3, C4, C6, C10, C12, C12:1, C14:1, C16, C18:1, and C18:2-acylcarnitine). Cirrhotic symptom score was significantly correlated with C0, C2, C3, C16, and C18–1-acylcarnitine blood levels. Among the 36-item short-form health survey (SF-36) items, the physical component summary was significantly associated with C0, C2, and C18–1-acylcarnitine blood levels.

**Conclusions:**

Carnitine fraction levels were positively correlated with liver cirrhosis stage, particularly, long-chain acylcarnitines. Moreover, carnitine fraction levels were associated with various subjective physical symptoms in liver cirrhosis patients.

## Background

Carnitine is known to be an important biofactor in fatty acid oxidation. Carnitine is essential for the transport of long-chain fatty acids from cytoplasm to mitochondria. The acylcarnitine in which an acyl-base derived from long-chain fatty acid and carnitine are ester-linked is transported into mitochondria, where acylcarnitines are converted to acyl-CoA at the inner mitochondrial membrane, and are provided as the substrate for β-oxidation. Moreover, it eliminates intracellular acyl-compounds, regulating the ratio of coenzyme A (CoA) to acyl-CoA [1, 2].

Blood carnitine level could be influenced by various metabolic states, such as liver cirrhosis, end stage renal failure, and type 2 diabetes [3–6]. Administration of L-carnitine is used for the treatment of cirrhosis-related symptoms, such as muscle cramps and hepatic encephalopathy [4, 7].

Acylcarnitine analysis using tandem mass spectrometry (MS/MS) has been developed as a test for fatty acid oxidation disorders, congenital defect in mitochondrial fatty acid oxidation enzymes, in pediatric field.

Carnitine exists as free carnitine and acylcarnitine fractions in blood samples; the acylcarnitine fraction can be further subdivided into multiple compounds. The amount of each acylcarnitine fraction in a blood sample was too small to accurately measure until recently. Advances in technologies, such as MS/MS, have enabled detailed analyses of acylcarnitine fractions in blood; however, there have been few studies on the factors associated with blood acylcarnitines in patients with liver cirrhosis [8].

In this study, we measured the free carnitine and acylcarnitines in patients with liver cirrhosis using MS/MS and compared the results with data from other laboratory tests and questionnaires.

## Methods

### Study population, design, and ethics statement

Fifty-four patients (median 67 years; male:female 34:20) with liver cirrhosis being treated at Nagasaki University Hospital were enrolled in this study. Twenty-four patients had hepatocellular carcinoma (HCC). We diagnosed liver cirrhosis based on clinical and laboratory data or histologic examination of liver biopsy specimens. Patients’ clinical characteristic are shown in Table 1.

**Table 1:** Patients’ clinical characteristics (*n* = 54)

Age, years (median, range)	67 (32–84)
Gender, male/female	34/20
Child-Pugh score	7 (5–14)
Body mass index (kg/m^2^)	23.5
Etiology HBV/HCV/AH/NASH/others	5/14/17/6/13
HCC +/−	24/30
eGFR	68.0 (34–126)

Abbreviations: *HBV* hepatitis B virus; *HCV* hepatitis C virus; *AH* alcoholic hepatitis; *NASH* non-alcoholic steatohepatitis; *HCC* hepatocellular carcinoma; *eGFR* estimated glomerular filtration rate

Six patients received a protein diet restriction (range 30 to 50 g daily) and 12 patients received a sodium restricted diet (6 mg daily).

Free carnitine (C0) and each acylcarnitines (i.e. C2, C4, C5, C6, C8, C8:1, C10, C12, C12:1, C14:1, C16, C18, C18:1,and C18:2) in a dried blood spot (DBS) were measured by using dried blood spotDBS with MS/MS in accordance with standardized protocols of non-derivatized method using NeoBase kit (Perkin Elmer, MA, USA). In brief, a single 3 mm DBS punch was placed in each well of 96-well assay plate, and 100 μL of the extraction solution containing internal standard of acylcarnitines and amino acids was added to each well. The plate was shaken at 700 rpm at 45 °C for 45 min, and the supernatant was transferred to another plate after the plate was centrifugation for 5 min at 1000×g. MS/MS analyses was performed by flow injection. Samples were measured using a Nexera MP System utilizing the SIL-30ACMP Multi-Plate autosampler and LCMS-8040 triple quadrupole mass spectrometer (Shimadzu Corporation, Kyoto, Japan) with an ESI positive source. The mobile phase was provided using the NeoBase Kit. The main component of the mobile phase was methanol. Injection volume was 1 μL, and total analytical time was 1 min.

The measurements were evaluated by using reference values optimized for clinical testing. The levels of free carnitine and acylcarnitines were assessed correlation of clinical factors, such as age, gender, body mass index (BMI), blood ammonia level, Child-Pugh score, HCC, and estimated glomerular filtration rate (eGFR), which were previously reported to be correlated with carnitine levels [6, 8].

Written informed consent was obtained from all patients. This study was approved by the ethics committee of Nagasaki University (No, 17022735).

### Questionnaires

The cirrhosis-related symptom scores (CSS) questionnaire, which we developed, was used to evaluate cirrhosis symptoms [9]. The Epworth Sleepiness Scale (ESS) was used to evaluate daytime hypersomnolence [10]. Sleep quality was assessed using the Japanese version of the Pittsburgh Sleep Quality Index (PSQI) [11]. Health-related quality of life was evaluated using the Japanese 36-item short-form health survey (SF-36) [version 2; Medical Outcomes Trust (Hanover, NH, USA), Health Lab (Hanover, NH, USA), QualityMetric (Lincoln, RI, USA), and Shunichi Fukuhara (iHope International; Kyoto, Japan)].

### Statistical analysis

We analyzed all data using SPSS version 20.0 software (SPSS, Chicago, IL, USA) and *P* < 0.05 was considered statistically significant. Discrete variables are presented as means with ranges. Laboratory data were analyzed using t-tests or chi-square tests, as appropriate. Correlations were determined using Pearson’s linear regression analysis. A multivariate analysis was performed using binary logistic regression analysis.

## Results

### Acylcarnitine analyses in cirrhotic patients

Average levels of free carnitine (C0) and acylcarnitines in dried blood spot of cirrhotic patients are shown in Table 2.

**Table 2:** Carnitine fraction levels (mean ± s.d.) (Standard values)

C0 (Free carnitine) mmol/L	50.4 ± 20.8 (20~70)
C2-acylcarnitine mmol/L	24.1 ± 12.1 (5~45)
C3-acylcarnitine mmol/L	3.1 ± 1.7 (~ 3.5)
C4-acylcarnitine mmol/L	0.23 ± 0.11 (~ 1.4)
C5-acylcarnitine mmol/L	0.25 ± 0.67 (~ 0.7)
C6-acylcarnitine mmol/L	0.10 ± 0.04 (~ 0.15)
C8-acylcarnitine mmol/L	0.10 ± 0.05 (~ 0.3)
C8–1-acylcarnitine mmol/L	0.15 ± 0.09 (~ 0.3)
C10-acylcarnitine mmol/L	0.12 ± 0.07 (~ 0.25)
C12-acylcarnitine mmol/L	0.05 ± 0.03 (~ 0.3)
C12–1-acylcarnitine mmol/L	0.12 ± 0.05 (~ 0.3)
C14–1-acylcarnitine mmol/L	0.10 ± 0.04 (~ 0.3)
C16-acylcarnitine mmol/L	2.0 ± 0.9 (0.4~3.0)
C18-acylcarnitine mmol/L	0.76 ± 0.54 (~ 2.0)
C18–1-acylcarnitine mmol/L	2.8 ± 1.4 (~ 2.8)
C18–2-acylcarnitine mmol/L	0.57 ± 0.30 (~ 0.8)

**Fig. 1:**
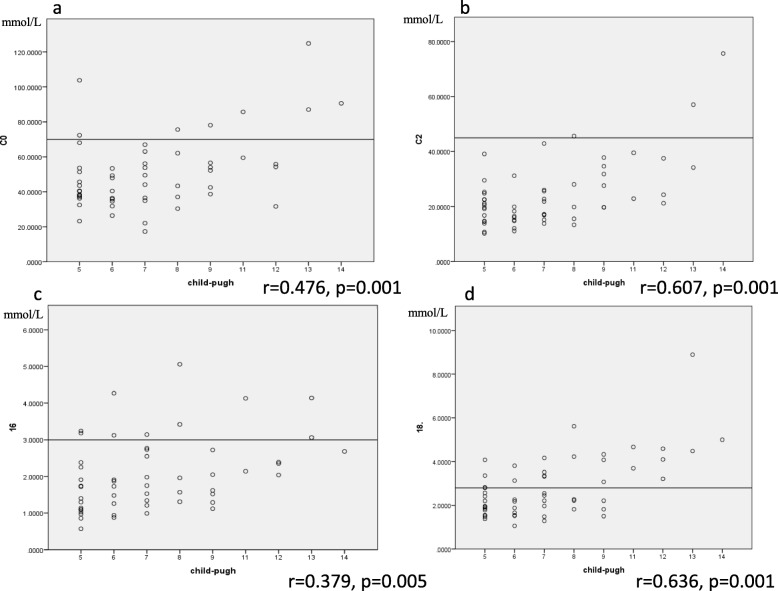
The Child-Pugh score is significantly correlated with the blood level of C0 (**a**), C2 (**b**), C-16 (**c**), and C18–1 (**d**). r = correlation coefficient. Each reference quantity is shown as a transverse line

In most cirrhotic patients, C0 (46 patients) and C2 (51 patients) values were within the normal range. Some patients had C16 (10 patients) and C18–1 (23 patients) levels higher than the normal range. The Child-Pugh score was significantly correlated with blood levels of C0, C2, C3, C4, C6, C10, C12, C12:1, C14:1, C16, C18:1, and C18:2 (Table 3). C18:1 level was high in all patients with a Child-Pugh score of more than 11 (Fig. 1a, b, c, d). Blood ammonia level was significantly correlated with C0 (r = 0.363, *p* = 0.011) and C8 levels (r = 0.355, *p* = 0.014).

**Table 3:** The correlation with Child-Pugh score and carnitine fraction level

	Correlation Coefficient	*P*-value
C0 (Free carnitine)	0.476	0.001
C2-acylcarnitine	0.607	0.001
C3-acylcarnitine	0.276	0.043
C4-acylcarnitine	0.357	0.009
C5-acylcarnitine	0.114	0.413
C6-acylcarnitine	0.294	0.031
C8-acylcarnitine	0.139	0.319
C8–1-acylcarnitine	−0.086	0.534
C10-acylcarnitine	0.356	0.008
C12-acylcarnitine	0.414	0.002
C12–1-acylcarnitine	0.486	0.001
C14–1-acylcarnitine	0.495	0.001
C16-acylcarnitine	0.379	0.005
C18-acylcarnitine	0.162	0.246
C18–1-acylcarnitine	0.636	0.001
C18–2-acylcarnitine	0.571	0.001

### Factors associated with acylcarnitines

The acylcarnitines found in the highest quantities in blood (i.e. C0, C2, C3, C16, and C18–1) were analyzed for associations with other clinical parameters. In univariate analyses, C0 or C3 levels were significantly correlated with age and Child-Pugh score, C2 or C18–1 levels were significantly correlated with only Child-Pugh score, and C16 level was significantly correlated with eGFR and Child-Pugh score (Table 4).

**Table 4:** Association between clinical factors and carnitine fraction level

		C0	C2	C3	C16	C18.1
Age	Correlation Coefficient	−0.314	−0.239	−0.386	−0.258	−0.221
	P-value	0.021	.0082	0.004	0.059	0.108
BMI	Correlation Coefficient	−0.243	−0.237	−0.148	− 0.106	−0.131
	P-value	0.079	0.087	0.290	0.448	0.351
eGFR	Correlation Coefficient	−0.054	−0.138	0.107	0.282^*^	0.012
	P-value	0.700	0.321	0.442	0.039	0.931
CPS	Correlation Coefficient	0.476	0.607	.0276	0.379	0.636
	P-value	0.001	0.001	0.043	0.005	0.001

Abbreviations: *BMI* body mass index; *eGFR* estimated glomerular filtration rate; *CPS* Child-Pugh score

There was no significant difference in each acylcarnitine level by etiology of liver cirrhosis. In multivariate analyses, C0 level was significantly associated with BMI, age, and Child-Pugh score whereas C2 level was significantly associated with BMI and Child-Pugh score. C16 and C18–1 levels were significantly associated with Child-Pugh score and eGFR.

### Questionnaire data associated with carnitine fractions

CSS scores were significantly associated with the levels of C0, C2, C3, C16, and C18–1. Scores on sleep questionnaires, such as the PSQI and ESS, were not significantly correlated with levels of any carnitine fraction. On the SF-36 questionnaire, the physical component summary score was significantly associated with the levels of C0, C2, and C18–1; however, the score of the mental component summary showed no significant correlation with any carnitine fraction level (Table 5).

**Table 5:** The correlation with questionnaire data and carnitine fraction level

			C0	C2	C3	C16	C18.1
CSS		Correlation Coefficient	.314^*^	.286^*^	.281^*^	.379^**^	.336^*^
		*P* value	.026	.044	.048	.007	.017
PSQI		Correlation Coefficient	.148	.086	.166	.048	.188
		P value	.312	.557	.253	.741	.195
ESS		Correlation Coefficient	−.075	.033	.114	.141	.036
		P value	.611	.822	.434	.334	.807
SF-36	PCS	Correlation Coefficient	−.355^*^	−.486^**^	−.083	−.262	−.403^**^
		P value	.016	.001	.583	.078	.005
	MCS	Correlation Coefficient	−.171	−.191	−.179	−.116	−.207
		P value	.255	.203	.233	.443	.168
	RCS	Correlation Coefficient	−.100	−.175	−.157	−.169	−.243
		P value	.508	.246	.297	.262	.103

Abbreviations: *CSS* cirrhosis-related symptom scores; *PSQI* Pittsburgh Sleep Quality Index; *ESS* Epworth Sleepiness Scale; SF-36, 36-item short-form health survey; health; *PCS* Physical component summary; *MCS* Mental component summary; *RCS* Role/Social component summary

## Discussion

In this study, we showed that levels of C0 and acylcarnitines in blood are significantly associated with disease measures in liver cirrhotic patients. The blood levels of most carnitine fractions showed a significant correlation with Child-Pugh score. This result is contrary to a previous study [4]. Shiraki et al. reported that Child-Pugh score was not significantly correlated with total carnitine, free carnitine, or acylcarnitine in cirrhotic patients [4]. The discrepancy may be due to differing methods for measuring carnitine. Shiraki et al. used the enzyme cycling method, which is not able to measure each acylcarnitines, whereas we used the LC-MS/MS method, which can accurately measure each carnitine fraction in blood.

Carnitine dynamics in cytoplasm via carnitine-Palmitoyl-Transferase-1 (CPT-1) is involved in the lipid metabolism in the liver [1, 2]. In this study, we measured carnitine profiling in dried blood sample. Although dried blood sample contains a red blood cell, plasma does not contain the red blood cell component, which means that the measurement in dried blood cell reflects carnitine profiling in cell cytoplasm.

Cirrhosis patients are known to be in a metabolic state similar to starvation [12]. In this study, all patients’ blood was sampled 12 h after dinner. This corresponds to 48 h of starvation in normal subjects [13]. We found the C2 acylcarnitine, which is the major carnitine fraction upregulated in starvation [14], was elevated in cirrhosis patients. Changes in lipid metabolism and branched-chain amino acid metabolism are known to occur in patients with advanced cirrhosis [15]. These metabolic changes in advanced cirrhosis patients may involve elevated levels of certain carnitine fractions.

In this study, their diets and dairy activities were variable. With advanced cirrhosis levels, patients tend to receive a restricted diet and dairy activities. These factors can influence on levels of carnitine fractions.

The free carnitine and short-chain acylcarnitines were within standard reference ranges in the majority of liver cirrhosis patients; however, long-chain acylcarnitines, such as C16 and C18–1, were higher than standard reference ranges in cirrhotic patients and significantly correlated with Child-Pugh score. In the liver, most of the cellular ATP is provided by fatty acid oxidation [16]. Long-chain acylcarnitine has an important role in shuttling long-chain fatty acids into mitochondria [17]. Accumulation of long-chain acylcarnitine suggests that more fatty acids can enter mitochondria [18]. The predominant fuel for ATP production shifts from glucose to lipid oxidation in patients with liver cirrhosis. In advanced liver disease, blood free fatty acid levels increase. Long-chain acylcarnitine levels in patients with diabetes showed a significant correlation with free fatty acid [5]. A similar mechanism may be occurring in liver cirrhosis patients. The increased level of long-chain acylcarnitine could be the marker of nutrition status in patient with advanced liver cirrhosis.

Some carnitine fractions levels are associated with age, BMI, renal function. These factors are affected by muscle volume. Carnitine is distributed skeletal muscle. These results may reflect muscle mass volume.

Neither sleep disturbance by the ESS and PSQI nor mental summary scores, assessed by the SF-36 questionnaire, showed any correlation with carnitine fraction levels. On the other hand, liver cirrhosis-related symptom scores (CSS questionnaire) and the physical component summary score on the SF-36 were significantly associated with the levels of carnitine fractions. These results indicate that carnitine fractions can be associated with physical symptoms in cirrhotic patients. Liver cirrhosis patients display various symptoms of energy shortage. Supplementation with carnitine is effective for improving symptoms, such as general fatigue, muscle cramps, and hepatic encephalopathy [4, 7, 18, 19]. Carnitine fraction can be the marker of the supplementation with carnitine.

Zhou et al. reported that long-chain carnitines, such as C16 and C18, accumulated to varying degrees in different liver diseases: with the lowest accumulation in chronic hepatitis, then liver cirrhosis, and the highest accumulation in HCC [8]. In our study, multivariate analysis demonstrated that these long-chain carnitines had no significant association with liver cirrhosis or HCC. This difference may be due to the relatively good liver function in HCC patients in this study.

This study has some limitations. First, this study was a small, retrospective study. Additional studies with larger sample sizes are needed to validate this study. Second, this study only enrolled liver cirrhosis patients. Ideally, we would compare liver cirrhosis patients with control subjects. In addition, this study was a cross-sectional study; therefore, we were not able to assess changes in the levels of carnitine fractions over time.

## Conclusion

Elevated carnitine fraction levels significantly correlated with increasing grade of liver cirrhosis, as quantified by Child-Pugh score. This association was particularly strong for long-chain acylcarnitines. Moreover, carnitine fraction levels were also significantly associated with various subjective physical symptoms in liver cirrhosis patients.

## Data Availability

The datasets during and/or analysed during the current study available from the corresponding author on reasonable request.

## Abbreviations

BMI: Body mass index
CoA: Coenzyme A
CSS: Cirrhosis-related symptom scores
EGFR: Estimated glomerular filtration rate
ESS: Epworth Sleepiness Scale
HCC: Hepatocellular carcinoma
MS/MS: Tandem mass spectrometry
PSQI: Pittsburgh Sleep Quality Index
SF-36: 36-item short-form health survey
